# New Approaches to Treatment of Severe Intrauterine Growth Restriction

**DOI:** 10.5195/cajgh.2014.166

**Published:** 2014-12-12

**Authors:** Zhanar Kurmangali

**Affiliations:** 1 National Research Center for Maternal and Child Health, Astana, Kazakhstan; 2 Center for Life Sciences, Nazarbayev University, Astana, Kazakhstan

**Keywords:** intrauterine growth restriction, pregnancy, fetal growth

## Abstract

**Introduction:**

Intrauterine growth restriction (IUGR) is a leading cause of perinatal morbidity and mortality due to placental insufficiency. Currently, one of the new approaches to treating this disease is the injection of nutrients to the fetus through intravascular port-systems (catheters).

**Objective:**

To assess the impact of nutrient injections as treatment to fetuses with severe growth retardation.

**Materials and methods:**

Pregnant women with IUGR (abdominal circumference (AC) < 5th percentile) with the absence of diastolic flow in the umbilical artery and a fetal gestational age of less than 30 weeks were randomly divided into two groups. The treatment group included six pregnant women who had an intravascular port-system for the infusion of nutrients (amino acids and glucose) in the umbilical vein of the fetus for 14 ± 3 days. The control group consisted of eight patients who received only traditional dynamic monitoring and delivery at the optimum time of pregnancy. Fetal status was assessed using ultrasound equipment Accuvix V20 (Medison, South Korea) by examining indicators of biometry and Doppler study of blood flow in utero, umbilical arteries, middle cerebral artery, and ductus venosus with fetal vascular resistance index calculation - pulsatility index (PI). Criteria for blood flow disturbances in the vessels were considered PI values above normal values for their gestational age, which were defined as absence or reverse blood flow in a diastole in the umbilical artery.

**Results:**

In a comparative analysis of the two groups, the treatment led to a 44.7% increase in AC of the fetus (121.0 ± 11.5 mm and 219.3 ± 18.3 mm, respectively, *p* < 0.001). In all cases, the profile of blood flow in the umbilical artery had a positive diastolic component. As a result, there was a 45.3% decrease in PI in the umbilical artery (2.14 ± 0.54 and 1.17 ± 0.15, respectively, *p* < 0.05). Average fetal weight in the study group was not significantly higher than the control group (1,120.3 ± 213.6 g and 909.6 ± 131.4 g, *p* > 0.05).

**Conclusion:**

Thus, injection of nutrients to the fetuses through intravascular port-system improved placental perfusion and metabolism, which has the potential for improved fetal growth. This, in turn, promoted full-term pregnancy and improved perinatal outcomes in fetal pathology.

